# MS4A superfamily molecules in tumors, Alzheimer’s and autoimmune diseases

**DOI:** 10.3389/fimmu.2024.1481494

**Published:** 2024-12-09

**Authors:** Xuejiao Luo, Bin Luo, Lei Fei, Qinggao Zhang, Xinyu Liang, Yongwen Chen, Xueqin Zhou

**Affiliations:** ^1^ Department of Dermatology, The Affiliated Hospital of the Non-Commissioned Officer (NCO) School, The Army Medical University, Shijiazhuang, Hebei, China; ^2^ Institute of Immunology, Department of Basic Medicine, The Army Military Medical University, Chongqing, China; ^3^ Chronic Disease Research Center, Medical College, Dalian University, Dalian, Liaoning, China; ^4^ Department of Otolaryngology, The Second Affiliated Hospital of the Army Military Medical University, Chongqing, China

**Keywords:** MS4A, Alzheimer’s disease, autoimmune diseases, tumors, immunotherapy

## Abstract

MS4A (membrane-spanning 4-domain, subfamily A) molecules are categorized into tetraspanins, which possess four-transmembrane structures. To date, eighteen MS4A members have been identified in humans, whereas twenty-three different molecules have been identified in mice. MS4A proteins are selectively expressed on the surfaces of various immune cells, such as B cells (MS4A1), mast cells (MS4A2), macrophages (MS4A4A), Foxp3^+^CD4^+^ regulatory T cells (MS4A4B), and type 3 innate lymphoid cells (TMEM176A and TMEM176B). Early research confirmed that most MS4A molecules function as ion channels that regulate the transport of calcium ions. Recent studies have revealed that some MS4A proteins also function as chaperones that interact with various immune molecules, such as pattern recognition receptors and/or immunoglobulin receptors, to form immune complexes and transmit downstream signals, leading to cell activation, growth, and development. Evidence from preclinical animal models and human genetic studies suggests that the MS4A superfamily plays critical roles in the pathogenesis of various diseases, including cancer, infection, allergies, neurodegenerative diseases and autoimmune diseases. We review recent progress in this field and focus on elucidating the molecular mechanisms by which different MS4A molecules regulate the progression of tumors, Alzheimer’s disease, and autoimmune diseases. Therefore, in-depth research into MS4A superfamily members may clarify their ability to act as candidate biomarkers and therapeutic targets for these diseases. Eighteen distinct members of the MS4A (membrane-spanning four-domain subfamily A) superfamily of four-transmembrane proteins have been identified in humans, whereas the MS4A genes are translated into twenty-three different molecules in mice. These proteins are selectively expressed on the surface of various immune cells, such as B cells (MS4A1), macrophages (MS4A4A), mast cells (MS4A2), Foxp3^+^CD4^+^ regulatory T cells (MS4A4B), type 3 innate lymphoid cells (TMEM176A and TMEM176B) and colonic epithelial cells (MS4A12). Functionally, most MS4A molecules function as ion channels that regulate the flow of calcium ions [Ca^2+^] across cell membranes. Recent studies have revealed that some MS4A proteins also act as molecular chaperones and interact with various types of immune receptors, including pattern recognition receptors (PRRs) and immunoglobulin receptors (IgRs), to form signaling complexes, thereby modulating intracellular signaling and cellular activity. Evidence from preclinical animal models and human genetic studies suggests that MS4A proteins play critical roles in various diseases (2). Therefore, we reviewed the recent progress in understanding the role of the MS4A superfamily in diseases, particularly in elucidating its function as a candidate biomarker and therapeutic target for cancer.

## 1 MS4A genes and structural characteristics of MS4A proteins

The human MS4A superfamily consists of eighteen different members (MS4A115 and MS4A18), which are encoded by genomic loci located on chromosome 11q12, in contrast, the MS4A genes in mice are located on chromosome 19 and encode twenty-three different molecules (1). TMEM176A (HCA112) and TMEM176B (LR8/TORID), whose genes are specifically located at 7q36.1 on chromosome 7, are also categorized as members of the MS4A superfamily because they have similar four-transmembrane protein structures, but their proteins only share ~16% amino acid sequence similarity with MS4A molecules (3). Genome-wide association studies (GWAS) have identified susceptibility genes for atopy and allergic diseases within this genomic region, indicating that mutations in *MS4A* genes might predispose individuals to related diseases (4). The *MS4A* genes first appeared in cartilaginous fish and are expressed in tissues beyond the immune system. These genes have since evolved and are now present in various vertebrates, including mammals, birds, reptiles, and amphibians. The *TMEM176* gene, however, is expressed primarily in mammals and bony fish (3).

Most MS4A proteins consist of 200~300 amino acids, with a molecular weight of approximately 22~35 kDa. According to transmembrane prediction databases, nearly all identified MS4A proteins, except for MS4A4E and MS4A6E, possess a four-transmembrane structure, therefore, they are classified as tetraspanins (2). Tetraspanins can interact with other proteins to form tetraspanin-enriched microdomains (TEMs) on the cellular membrane, which play critical roles in regulating cellular physiological processes (5). MS4A molecules typically contain two extracellular loops and one intracellular loop, and both the N- and C-termini are located intracellularly. Among the different MS4A proteins, the first three transmembrane regions exhibit high homology: the first transmembrane region of the extracellular loop has approximately thirteen amino acids and consists of conserved amino acid sequences such as VLGAIQIL, LGAXQI, and LSLG. The second extracellular loop varies in length from 1046 amino acids, with its transmembrane region containing conserved sequences, such as GYPFWG and FIISGSLS, and this region exhibits significant heterogeneity between different molecules. Interestingly, the conserved sequences SLX2NX2 and SX3AX2G are commonly found in the third transmembrane region (6, 7). In addition to MS4A8B and MS4A12, most MS4A molecules possess two cysteine residues in the second extracellular loop that can form disulfide bonds. Additionally, the intracellular segments of MS4A proteins generally contain SH2 and SH3 domain binding sites (7). Notably, the intracellular segment of MS4A2 includes an immunoreceptor tyrosine-based activation motif (ITAM), whereas MS4A8B and TMEM176B contain an immunoreceptor tyrosine-based inhibitory motif (ITIM), these structures facilitate the formation of signal transduction complexes. Therefore, MS4A proteins can regulate cytoskeletal remodeling, transcriptional responses, signal transduction cascades, and cellular differentiation (8).

## 2 Characteristic expression of MS4A molecules in cell subsets and tissues

Most MS4A molecules are expressed on the surface of immune cells. For example, MS4A1 (CD20) is expressed primarily on the membrane of precursors and mature B cells, but its expression is lost on plasma cells. The high-affinity IgE receptor (FcϵRI) is a tetramer that consists of one α subunit, one β subunit, and two γ subunits. MS4A2 (FcϵRβ) serves as the β subunit of both the high-affinity FcϵRI and the low-affinity IgG receptor (FcγRIII), which are predominantly expressed on the surface of mast cells and basophils (6). MS4A3 (HTm4) is selectively expressed on various myeloid and lymphoid progenitor cells within the hematopoietic system (1). MS4A5 (TETM4.1) is expressed on the surface of precursor monocytes (2). Although MS4A8B (L985P) has been detected in B-cell lines, such as BJAB, DAUDI, and SB, it is expressed primarily on ciliated cells of the bronchial mucosal epithelium (6, 7). MS4A12 is exclusively expressed in the mucosal epithelial cells of colonic tissue and in colon cancer cell lines (9). Notably, TMEM176A (HCA112) and TMEM176B (LR8 or TORID) are highly expressed on immature or resting dendritic cells (DCs), as well as on the surface of helper T cells and type 3 innate lymphoid cells (ILC-3) (10). Moreover, MS4A4B appears to be restricted to Foxp3^+^ regulatory T cells (Tregs) based on a yeast split-ubiquitin Treg library screen (11). Notably, some MS4A proteins may exist as homo- and/or heterodimers, for example, TMEM176A and TMEM176B have been shown to interact with each other in DCs. Additionally, MS4A4A associates with itself and/or with two other members of the MS4A family, including MS4A6A and MS4A7, to form characteristic molecular clusters on the surface of macrophages (12). Here, we list the distributions of several major MS4A molecules in cell types and tissues (**Table 1**).

**Table 1:** Distribution and physiological functions of MS4A molecules.

MS4A	Distribution	Biofunctions	Tumor types	Other associated diseases	References
MS4A1	Tonsil, lymph nodes, bone marrow, spleen, amygdala, CD20^+^ B cells, T_RM_-cells, olfactory sensory neurons, mast cells, Natural killer-like B cells, etc.	Protein tetramerization, mammalian olfactory receptor, store-operated Ca^2+^ entry, Calcium channel protein of B lymphocytes, participating in differentiation B-cell differentiation, proliferation and activation	colorectal cancer, breast Cancer, intrahepatic cholangiocarcinoma, non-small cell lung cancer, lung adenocarcinoma, B-cell lymphoma, ovarian cancer, stomach adenocarcinoma, head and neck squamous cell carcinoma, nasopharyngeal carcinoma	Common Variable Immunodeficiency (CVID), rheumatoid arthritis, lupus nephritis, IV infection of rhesus macaques, *Mycobacterium tuberculosis* lung infection	(13–21)
MS4A2	Adrenal cortex, pituitary, thyroid, liver, heart, bone marrow, skin, brain, testis, skeletal muscle, cardiac myocytes, monocytes, Mast cells, CD34^+^ cells, CD33^+^ myeloid, etc.	Lipid metabolism, store-operated Ca^2+^ entry, high affinity for lgE receptor subunits of mast cells, involving in allergic reactions induced by allergens.	colorectal cancer, lung adenocarcinoma, gastric cancer	atopic dermatitis, type 2 diabetic peripheral neuropathy, Idiopathic Pulmonary Fibrosis, COVID-19, RA, osteoarthritis, preterm infants with congenital respiratory diseases, severe asthma patients, type 2 diabetes patients	(22–25)
MS4A3	CD34^+^ myeloid precursors, CD33^+^ myeloid, macrophage, dendritic cells; bone marrow, etc.	enhancing β-chain cytokine receptor endocytosis, binding with CDKN3/KAP regulates phosphorylation of CDK2 and G1-S transition, perinuclear region of cytoplasm.	chronic myeloid leukemia, ovarian cancer, breast cancer	Hematopoietic disorder, pregnancies, type 1 diabetic patients, murine coronavirus encephalomyelitis	(26–29)
MS4A4A	Lung, placenta, small intestine, CD33^+^ myeloid, macrophage, mast cells; bone marrow, etc.	store-operated Ca^2+^ entry, suspected to be related to development and secretion function of Th1 cells	Glioblastoma, mucinous colorectal adenocarcinoma, Lymphoma, gastric cancer, esophageal cancer, ovarian cancer, glioma, ovarian cancer, breast cancer, lung adenocarcinoma, gastric cancer	AD, diabetic nephropathy, pediatric sepsis, RA-associated interstitial lung disease, atherosclerosis, diabetic kidney disease	(30–34)
MS4A4E	Peripheral blood, spleen, liver, skin, etc.	No relevant functions reported	Glioma	AD	(34, 35)
MS4A5	Testes, CD33^+^ myeloid	Cell surface receptor signaling pathway	No relevant tumors reported	No relevant diseases reported	
MS4A6A	BDCA4^+^ DCs, CD14^+^ monocytes, CD68^+^ macrophages, CD33^+^ myeloid, CD105^+^ endothelial cells, etc.	Cell surface receptor signaling pathway	Glioma, glioblastoma, non-small cell lung cancer, lung adenocarcinoma, esophageal cancer, ovarian cancer	AD pathology, nonalcoholic fatty liver disease, periodontitis, and type 2 diabetes mellitus, lupus nephritis, chronic gastritis and osteoporosis, obesity, Kawasaki disease, multiple sclerosis, small vessel ischemic disease	(36, 37)
MS4A6E	Lymph nodes, testis, etc.	Cell surface receptor signaling pathway	No relevant tumors reported	polycystic ovary syndrome	(38–40)
MS4A7	Macrophage, peripheral blood, spleen, etc.	Suspected to be related to differentiation of mononuclear cells	glioblastoma, non-small cell lung cancer, Gastric Cancer, lung adenocarcinoma, esophageal cancer, Glioma	peripheral neuropathic pain No relevant diseases reported	(41–44)
MS4A8B	Colon, lung, trachea, skeletal muscle, prostate, testis, small intestine, etc.	Suspected to be related to proliferation of prostate cancer cells	Prostate cancer	No relevant diseases reported	(45)
MS4A10	Kidney, tonsil, lymph nodes, bone marrow, adrenal grands, small intestine, BDCA4^+^ DCs, CD14^+^ monocytes, CD68^+^ macrophages, CD33^+^ myeloid, etc.	No relevant functions reported	gastric cancer, metastatic colorectal cancer, primary colorectal cancer	patient with transient hyperCKemia and myalgia	(46–48)
MS4A12	Colon, pituitary.	Calcium channel protein on colonic cells, suspected to be related to proliferation of colon cancer cells	colon cancer	No relevant diseases reported	(48, 49)
MS4A13	Testes	No relevant functions reported	No relevant tumors reported	No relevant diseases reported	(50)
MS4A14	Testes	No relevant functions reported	Gastric cancer, lung adenocarcinoma	No relevant diseases reported	(51–53)
MS4A15	Lung and salivary glands	calcium-restricted lipid remodeling, reprogramming energy metabolism	ovarian cancer, lung adenocarcinoma, gastric cancer	Ferroptosis resistance	(51, 52, 54, 55)
MS4A18	Testis and small intestine.	No relevant functions reported	No relevant tumors reported	No relevant diseases reported	
TMEM176A	Kidney, fetal liver, fetal lung, pancreatic islets, liver, retina, CD14^+^ monocytes, CD68^+^ macrophages, CD33^+^ myeloid, etc.	Ion channels, cation channels	pancreatic cancer, lung cancer, triple-negative breast cancer, hepatocellular carcinoma, glioblastoma, esophageal squamous cell cancer, colorectal cancer, glioma, gastric cancer, bladder cancer, metastatic colon cancer	Chronic spinal cord Injury, Kimura’s disease, sporadic Ménière’s disease patients, Behçet’s syndrome, acute myocardial infarction, neovascular age-related macular degeneration, carotid atherosclerotic plaques, negative regulation of dendritic cell differentiation.	(56–60)
TMEM176B	Kidney, fetal liver, fetal lung, colon, small intestine; pancreatic islets, liver, retina, CD14^+^ monocytes, CD68^+^ macrophages, CD33^+^ myeloid, whole blood, etc.	intracellular cation channel, unleashing inflammasome activation, amino acid metabolism, regulation of myogenic differentiation; development of DCs and cerebellar granule cells	Triple-negative breast cancer, non-small cell lung cancer, lung adenocarcinoma, colorectal cancer, skin cutaneous melanoma, prostate cancer, gastric cancer, diffuse large B-Cell lymphoma, colorectal cancer	Chronic spinal cord injury, acute respiratory distress syndrome, nonclassical monocytes, atrial fibrillation; negative regulation of dendritic cell differentiation.	(58, 61–63)

## 3 The predominant biofunctions of MS4A molecules

### 3.1 MS4A proteins act as ion channels

Early studies confirmed that MS4A molecules function primarily as ion channels that regulate the exchange of calcium ions [Ca^2+^] between the intracellular and extracellular environments. For example, MS4A1 is present in both homodimers and heterodimers on human B lymphoblastoid cells, where it serves as an ion channel to regulate [Ca^2+^] flow across the cell membrane, as evidenced by the increase in [Ca^2+^] conductance after transfection with MS4A1 (64, 65). MS4A1 crosslinks with BCR and positively regulates BCR-induced cytoplasmic [Ca^2+^] mobilization (66). MS4A2 contains an ITAM motif ((D/E)-XXYXXL-(X)7–9YXX-L/I) in its C-terminus, and crosslinking of the IgE-bound FcϵRI receptor by an antigen can phosphorylate tyrosine residues in the ITAMs of MS4A2, triggering downstream signal cascades that result in the mobilization of intracellular calcium stores (67); conversely, the downregulation of *MS4A2* reduces [Ca^2+^] influx upon IgE cross-linking (67). Human mast cells express MS4A4A, and silencing *MS4A4A via* RNAi reduces [Ca^2+^] influx upon IgE crosslinking, suggesting that MS4A4A can also regulate store-operated [Ca^2+^] entry (68). MS4A12 is a novel component of store-operated [Ca^2+^] entry in intestinal cells, and loss of *MS4A12* in LoVo colon cancer cells attenuates epidermal growth factor receptor-mediated signaling, thereby promoting colonic carcinoma migration (9). Recent two-photon imaging of olfactory epithelial cells has also demonstrated that different MS4A proteins can recognize specific chemical ligands, thereby increasing the activity of the calcium indicator GCaMP6 (69). Additionally, *Xenopus* oocytes express TMEM176B, which initiates an inward current activated by acidification of the extracellular solution to pH 5, suggesting that TMEM176B might function as an acid-sensitive, nonselective, monovalent cation channel (61). Because TMEM176A also induces an inward current activated by acidification of the extracellular solution in *Xenopus* oocytes, TMEM176A may act as a cation channel (70).

### 3.2 MS4A proteins function as molecular chaperones

**Figure 1:**
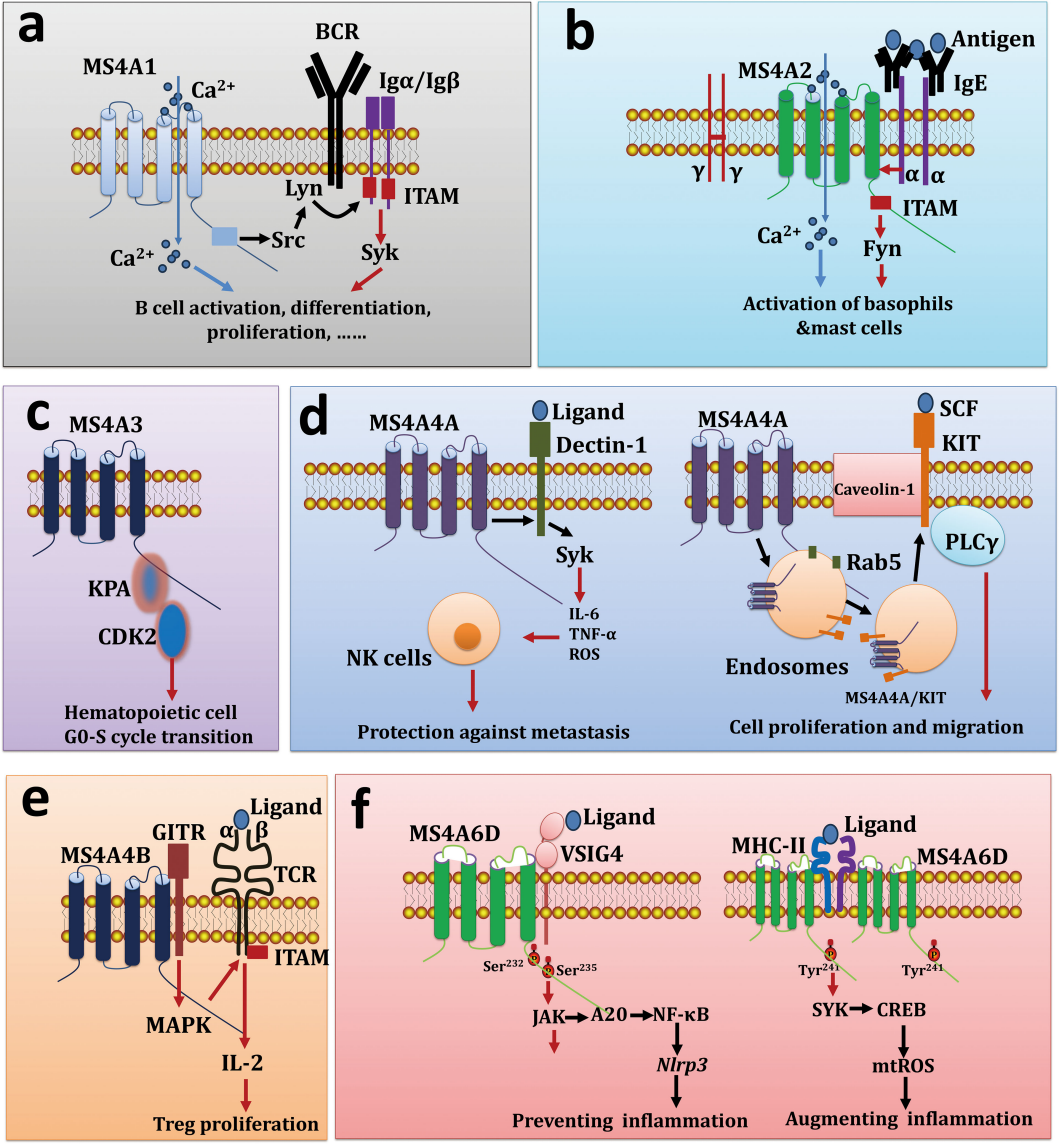
MS4A proteins function as molecular chaperones. **(A)** MS4A1 interacts with BCR to induce B cell activation, differentiation, and proliferation. **(B)** MS4A2 as a receptor for IgE and triggers the activation of basophils as well as mast cells. **(C)** MS4A3 promotes hematopoietic cell G0-S cycle transition via interaction with KPA and CDK2. **(D)** MS4A4A/Dectin-1 complex induce NK cell activation and protection against metastasis (left), on the other hand, MS4A4A/KIT complex also can controls cell proliferation and migration (right). **(E)** MS4A4B crosslink with GITR and control Treg proliferation. **(F)** VSIG4 forms a complex with MS4A6D, regulating the Jak-STAT1-A20-NF-κB pathway, which in turn influences the transcription of *Nlrp3* and its substrate *Il1b* genes (left), on the other hand, MHC-II forms a complex with MS4A6D, regulating the SYK-CREB-SDHB pathway, which affects mitochondrial mtROS secretion, leading to macrophage inflammatory responses.

Recent studies have suggested that some MS4A molecules function as chaperones that interact with pattern recognition receptors (PRRs) and/or immunoglobulin receptors (IgRs). For example, MS4A1 interacts with membrane proteins, such as major histocompatibility complex (MHC) class I proteins, MHC class II proteins, tetraspanins (CD53, CD81, and CD82), and the BCR, thereby promoting B-cell activation and antibody class switching (**Figure 1A**) (71, 72). MS4A2 associates with FcϵRIα and FcϵRIγ to form a tetrameric receptor complex (αβγ2) on mast cells at high density, therefore, MS4A2 acts as a signal amplifier through its ability to increase Lyn-dependent phosphorylation of FcϵRIγ (**Figure 1B**) (73). Human MS4A3 (HTm4) regulates the cell cycle of hematopoietic cells and controls their differentiation into various cell types through binding to the cyclin-dependent kinase-associated (CDK-associated) phosphatase-CDK2 (KAP-CDK2) complex (74), and the overexpression of MS4A3 causes cell cycle arrest at the G(0)/G(1) phase (**Figure 1C**). MS4A4A4A interacts with and colocalizes with the β-glucan receptor Dectin-1 in lipid rafts, and the MS4A4A/Dectin-1 complex can trigger downstream spleen tyrosine kinase (SYK) signaling cascades to induce macrophage and NK cell activation in response to dectin-1 ligands (12, 75). Additionally, MS4A4A on mast cells facilitates trafficking of the receptor tyrosine kinase KIT to caveolin-1-rich microdomains through endocytic recycling rather than degradation pathways, thus promoting KIT signaling in endosomes (**Figure 1D**) (76). MS4A4B interacts with the glucocorticoid-induced tumor necrosis factor receptor (GITR) on Foxp3^+^CD4^+^ Tregs, forming a membrane signaling complex (MS4A4B/GITR) that enhances IL-2 secretion in response to triggering with GITR ligands or anti-GITR Abs (**Figure 1E**) (11). In mice, MS4A4B is highly expressed on T cells and influences T-cell apoptosis by controlling the activity of caspases 3, 8 and 9. Conversely, knocking down *Ms4a4b* with siRNA or shRNA promotes apoptosis in naïve T cells or the T32 cell line, whereas the overexpression of *Ms4a4b* reduces EL-4 cell apoptosis (77, 78).

Our recent research revealed that MS4A6D, a novel member of the MS4A superfamily, is restrictively expressed in tissue-resident macrophages and DCs in mice. MS4A6D interacts with VSIG4 to form a cell surface signaling complex, activating the STAT3-A20-NF-κB signaling pathway and thereby inhibiting the transcription of the *Nlrp3* and *Il1b* genes in peritoneal macrophages (**Figure 1F**) (79). Recent studies have shown that MS4A6D on the surface of monocyte−macrophages cross-links with MHC-II molecules under inflammatory conditions, activating downstream SYK signaling and leading to the release of the inflammatory cytokines IL-1, IL-6, TNFα, and mitochondrial reactive oxygen species (mtROS) (80). Notably, we found that MS4A6D primarily exists as a homodimer, with the cysteine at position 237 playing a decisive role in dimer formation (**Figure 1F**). Therefore, MS4A6D plays a crucial role in macrophage activation and the progression of inflammatory diseases.

## 4 Associations between MS4A molecules and diseases

Evidence from preclinical animal models and human genetic studies indicates that most members of the MS4A superfamily play critical roles in various pathological disorders, including cancer, infectious diseases, and neurodegenerative diseases. As a result, some MS4A members might serve as candidate biomarkers and therapeutic targets for specific diseases.

### 4.1 MS4A superfamily molecules and Alzheimer’s disease

Alzheimer’s disease (AD) is the most common cause of dementia worldwide, with the prevalence continuing to grow in part because of the aging population. The progression of this neurodegenerative disease is characterized by two hallmark pathologies: β-amyloid (Aβ) plaque deposition and neurofibrillary tangles of hyperphosphorylated Tau (81). AD predominantly affects elderly individuals, especially those who are older than 85 years. Nearly 43% of elderly individuals are speculated to suffer from AD in 2050, creating a substantial burden on patients and society (82). Removing aggregated Aβ from the brains of symptomatic patients can slow the progression of AD, but the clinical benefit achieved in these trials has been modest, highlighting the need for a deeper understanding of the pathogenesis of disease mechanisms.

GWAS have revealed a correlation between mutations in the MS4A gene cluster, particularly the rs610932 and rs4938933 loci of the *MS4A4A* gene, which are more susceptible to AD (83, 84). Additionally, polymorphisms at the rs1562990 locus, which is located between the *MS4A4E* and *MS4A4A* genes, have also been suggested to be linked with AD susceptibility (85). Astrocytes and microglia affect AD progression by clearing pathological proteins, and single-nucleus RNA sequencing (snRNA-Seq) studies have shown that the *MS4A6A* gene in microglia affects Aβ plaque deposition and Tau protein phosphorylation (86). The triggering receptor expressed on myeloid cells 2 (TREM2), which can regulate microglial activation and Aβ phagocytic function, is found on the surface of microglia. Furthermore, single-nucleotide polymorphisms (SNPs) within the *MS4A* gene cluster, such as rs1582763, are associated with increased levels of soluble TREM2 (sTREM2) in cerebrospinal fluid; remarkably, sTREM2 can reduce AD risk and delay AD onset; conversely, the rs6591561 locus is linked to decreased sTREM2 levels, leading to increased AD risk (30). Additionally, the rs667897 locus within the MS4A gene cluster promotes the expression of the *MS4A6A* gene, thereby increasing AD risk (87). Therefore, a deeper understanding of the role of MS4A molecules in the pathogenesis of AD would help develop new preventive and therapeutic strategies.

### 4.2 MS4A superfamily molecules and autoimmune diseases

Autoimmune diseases (AuDs) are characterized by a loss of immune tolerance to self-antigens, leading to strong immune responses and thus resulting in tissue damage. AuDs can be classified into organ-specific and systemic autoimmune diseases. Organ-specific AuDs include Hashimoto’s thyroiditis, Graves’ disease, and myasthenia gravis, whereas systemic ADs include rheumatoid arthritis (RA), systemic lupus erythematosus (SLE), and Sjögren’s syndrome (SS) (88). Approximately 5%~8% of the global population is affected by AuDs.

Several members of the MS4A family are implicated in the progression of AuDs. For example, MS4A1 is expressed primarily on the surface of B cells, which exacerbates the progression of AuDs. Rituximab, a monoclonal antibody (mAb) that targets MS4A1, has been approved by the U.S. Food and Drug Administration (FDA) and the European Medicine Agency (EMA) for the treatment of RA in the clinic (89). Additionally, MS4A4A, a macrophage marker within the MS4A family, has also been detected in the synovial tissue of early RA patients (90). The MS4A2 molecule is the β subunit of FcϵRI, and mice with *Ms4a2* gene knockout are protected from both skin and systemic allergic reactions (91). Human MS4A2 is not only associated with allergies but also linked to an increased risk of allergic rhinitis (91). Recent studies have shown that MS4A4A expression is associated with cutaneous systemic sclerosis, polyangiitis, and Kawasaki disease (92, 93). Our research has also identified MS4A6D as a potential therapeutic target for various inflammatory diseases, including colitis and psoriasis (94, 95). Therefore, certain MS4A family members may serve as novel therapeutic targets or immune interventions for AuDs.

### 4.3 MS4A superfamily molecules and tumors

In addition to being involved in controlling the pathogenesis of AD and autoimmune disorders, MS4A molecules are also associated with the occurrence and progression of hematological malignancies. We reviewed the different MS4A molecules in certain solid tumors in much greater detail (**Table 1**).

#### 4.3.1 MS4A1

MS4A1 is a characteristic marker of B cells and is closely related to the treatment and prognosis of lymphomas and lymphocytic leukemias. Rituximab, a mAb against MS4A1, was approved for the treatment of relapsed B-cell lymphomas and relapsed non-Hodgkin’s lymphoma (96). Recently, MS4A1 has also been detected in other cancer tissues, including glioblastoma, mucinous colorectal adenocarcinoma, lymphomas, esophageal cancer, ovarian cancer, glioma, and lung adenocarcinoma, indicating that the therapeutic application of Rituximab may extend beyond B-cell lymphomas (97). Mechanistically, the antitumor activity of Rituximab is attributed primarily to the induction of apoptosis, complement-dependent cytotoxicity (CDC), and antibody-dependent cell-mediated cytotoxicity (ADCC). Recent studies have suggested that antibody-dependent cellular phagocytosis (ADCP) may be the predominant mechanism by which Rituximab clears cancer cells (98). However, because Rituximab is a chimeric human-mouse anti-MS4A1 mAb, it contains approximately 30% mouse-derived sequences, which can elicit a human anti-mouse immune response (HAMA) when it is introduced into the human body, this side effect significantly restricts its clinical application. To reduce the immunogenicity of the mouse-derived sequences in Rituximab, antibody humanization techniques, such as “complementarity-determining region (CDR) grafting” have been employed (99). This technique involves grafting the CDRs of mouse antibodies onto the framework regions of human antibodies, thereby reducing the immunogenicity associated with the mouse antibody framework. In addition to Rituximab, other therapeutic strategies that target MS4A1, such as CAR-T cell therapy and antibody−drug conjugates (ADCs), are under active development (100, 101). CAR-T cell therapies have shown promise in overcoming resistance in relapsed hematologic malignancies, with several companies and institutions currently conducting clinical trials.

#### 4.3.2 MS4A3

MS4A3 (HTm4) is expressed on bone marrow-derived macrophage precursors, DCs, and monocytes in peripheral blood, making it a key regulator of the cell cycle in hematopoietic cells. Abnormal expression of MS4A3 leads to increased kinase-associated phosphatase activity, causing cells to arrest in the G0/G1 phase. Recent studies have revealed a close association between MS4A3 and tumorigenesis, with significant differences in MS4A3 expression observed in prostate cancer, ovarian cancer, and breast cancer tissues compared with normal tissues (26–29). The transcription factor EVI-1 (ecotropic virus integration site 1) is highly expressed in myeloid leukemia, and research by Heller G et al. revealed that overexpression of EVI-1 in myeloid leukemia cells suppresses MS4A3 expression, thereby promoting tumor growth (102), suggesting a potential link between MS4A3 and tumor development.

#### 4.3.3 MS4A6A

MS4A6A (also known as CDA01, MS4A6, 4SPAN3, or CD20L3) is a prominent member of the MS4A gene family (103). MS4A6A is expressed on the surface of classical CD14^+^CD16^-^monocytes and M2 macrophages, with minimal expression on CD14^-^CD16^+^ M1 macrophages and CD14^+^CD16^-^transitional macrophages, suggesting that MS4A6A might play a critical role in tissue repair (36). MS4A6A is significantly expressed in lung-infiltrated macrophages and can serve as a prognostic marker for non-small cell lung cancer (NSCLC) (37). In lung adenocarcinoma, researchers have reported a positive correlation between MS4A6A expression and the infiltration of immune cells, such as macrophages and DCs, within the tumor microenvironment (37, 104). We also detected the expression of MS4A6A in tissues from breast ductal carcinoma *in situ* (DCIS), and multicolor fluorescence staining revealed that MS4A6A is expressed mainly in infiltrated CD68^+^ macrophages. Moreover, survival is better among DCIS patients with high levels of MS4A6A-positive cells than among patients with low numbers of these cells. Our study revealed that MS4A6A can function as a prognostic marker in various malignant tumors, including DCIS, because of its role in tumorigenesis and tumor immunity. However, the precise role of MS4A6A in cancer progression remains unclear, and the relationship between MS4A6A expression in tumor tissues and immune cell infiltration needs further investigation.

#### 4.3.4 MS4A7

MS4A7 (CFFM4) is expressed primarily in monocyte/macrophage-containing tissues, such as the spleen, liver, and lungs. Moreover, research indicates that MS4A7 is expressed in various cancer tissues, including glioblastoma, lung adenocarcinoma, esophageal cancer, and glioma. In triple-negative breast cancer (TNBC), MS4A7 has been identified as a prognostic factor, and a predictive model that incorporates the MS4A7, SPARC, and CD300C genes has demonstrated strong prognostic accuracy (105). In gastric cancer, low mRNA transcription levels of MS4A7 are associated with better overall survival, whereas high mRNA transcription levels of MS4A6A suggest a better prognosis (51). Functionally, the regulatory role of MS4A7 in the differentiation of monocytic leukemia cells may be related to the activation of the p38 MAPK pathway.

#### 4.3.5 MS4A8B

MS4A8B is a recently identified member of the MS4A family that has been implicated in cell differentiation and tumorigenesis. Its murine homolog, MS4A8A, is expressed on tumor-associated macrophages in breast cancer and melanoma. Overexpression of MS4A8A in the RAW264.7 cell line has been shown to increase breast tumor growth in a mouse model of breast cancer. Additionally, MS4A8B is expressed on the surface of intestinal epithelial cells, and its expression is elevated in colorectal cancer. Immunohistochemical studies demonstrated that MS4A8B was upregulated in small cell lung cancer, and its levels have also been linked to the progression of prostate cancer (106). Silencing *Ms4a8b* in prostate cancer cell lines leads to cell cycle arrest, suggesting that MS4A8B promotes G1/S cell cycle transition (71), conversely, the overexpression of MS4A8A has been shown to significantly reduce the proliferation and migration rates of mouse colorectal cancer cells (33). Clinical studies have also revealed significant differences in MS4A8B protein expression across benign prostate tissue, adjacent prostate cancer tissue, prostatic intraepithelial neoplasia, prostate carcinoma *in situ*, and metastatic prostate cancer lymph nodes. Research has revealed that MS4A8B protein expression is associated with tumor recurrence, Gleason scores, and proliferation indices (45), indicating that MS4A8B expression is related to postsurgical recurrence and metastasis in prostate cancer patients.

#### 4.3.6 MS4A12

MS4A12 is specifically expressed in colonic tissue, and early immunohistochemical studies revealed that MS4A12 is expressed exclusively in colorectal cancer cells, with no expression in adjacent stromal or nontumor epithelial cells. The rate of MS4A12 positivity in colorectal cancer is as high as 63%, and MS4A12 expression is regulated by the transcription factor caudal type homeobox 2 (CDX2), which can influence the proliferation and cell cycle of colorectal cancer cells (9). Drew J et al. analyzed six colorectal cancer and six colorectal adenoma tissue samples and reported significant differences in MS4A12 expression between normal colon tissue, inflammatory polyps, and colorectal cancer tissues, suggesting that MS4A12 expression may be related to the degree of colonocyte differentiation (107). Additionally, Dalerba P et al. reported that patients with negative MS4A12 expression had significantly reduced survival rates (108), indicating that the unique expression pattern of MS4A12 in the colon may make it a novel target for colorectal cancer immunotherapy.

#### 4.3.7 TMEM176A and TMEM176B

TMEM176A and TMEM176B are unique members of the MS4A family with distinct expression characteristics, and recent studies have improved our understanding of their structures, distribution patterns, biological functions, and associations with various clinical diseases, including cancer. TMEM176A and TMEM176B interact with each other and function as ion channels that play critical roles in regulating antigen cross-presentation in DCs (10). Abnormal DNA methylation of CpG islands in human *TMEM176A* and *TMEM176B* is associated with breast cancer development (109). In hepatocellular carcinoma (HCC) tissues, the transcription of the 5’ and 3’ introns of the gene that encodes human *TMEM176B* is significantly reduced (110). Additionally, the expression levels of TMEM176A and TMEM176B differ significantly between cancerous and normal tissues in breast cancer, lymphoma, skin cancer, and liver cancer, suggesting their potential as diagnostic markers for tumors (111). Knockdown of the *TMEM176A* gene has been shown to inhibit the proliferation, migration, and invasion of colorectal cancer cells (112), indicating a possible role for TMEM176A in the invasion and metastasis of colorectal cancer. In breast cancer cells, the expression of TMEM176B is crucial for AKT/mTOR signaling, angiogenesis, KRAS signaling, epithelial−mesenchymal transition (EMT), and the regulation of estrogen and interferon response genes (62), therefore, therapeutic antibodies that target TMEM176B may inhibit tumor cell proliferation. These findings suggest that TMEM176A and TMEM176B could serve as novel targets for immunotherapy in certain cancers.

## 5 Perspective

MS4A family proteins play crucial regulatory roles in cell growth, survival, and activation. These proteins function physiologically as ion channels or signal modulators of immune receptors, often existing as homomeric and heteromeric complexes within lipid raft microdomains. Several MS4A members have significant physiological functions in various diseases, including Alzheimer’s disease, autoimmune disorders, and cancer. Future research should focus on elucidating the molecular mechanisms of signal transduction mediated by MS4A, with particular emphasis on the biological functions of various splicing variants of MS4A proteins. Understanding the mechanisms of action of MS4A family molecules will have profound implications for a wide range of diseases, including malignant tumors.
